# Enhancement of 5-FU sensitivity by the proapoptotic rpL3 gene in p53 null colon cancer cells through combined polymer nanoparticles

**DOI:** 10.18632/oncotarget.13216

**Published:** 2016-11-08

**Authors:** Annapina Russo, Sara Maiolino, Valentina Pagliara, Francesca Ungaro, Fabiana Tatangelo, Alessandra Leone, Giulia Scalia, Alfredo Budillon, Fabiana Quaglia, Giulia Russo

**Affiliations:** ^1^ Laboratory of Biochemistry, Department of Pharmacy, University of Napoli Federico II, 80131 Napoli, Italy; ^2^ Laboratory of Drug Delivery, Department of Pharmacy, University of Napoli Federico II, 80131 Napoli, Italy; ^3^ Istituto Nazionale Tumori “Fondazione Pascale”-IRCCS, 80131 Napoli, Italy; ^4^ CEINGE-Biotecnologie Avanzate, 80145 Napoli, Italy

**Keywords:** 5-FU, p53, ribosomal protein rpL3, colon cancer, apoptosis

## Abstract

Colon cancer is one of the leading causes of cancer-related death worldwide and the therapy with 5-fluorouracil (5-FU) is mainly limited due to resistance. Recently, we have demonstrated that nucleolar stress upon 5-FU treatment leads to the activation of ribosome-free rpL3 (L3) as proapoptotic factor. In this study, we analyzed L3 expression profile in colon cancer tissues and demonstrated that L3 mRNA amount decreased with malignant progression and the intensity of its expression was inversely related to tumor grade and Bcl-2/Bax ratio. With the aim to develop a combined therapy of 5-FU plus plasmid encoding L3 (pL3), we firstly assessed the potentiation of the cytotoxic effect of 5-FU on colon cancer cells by L3. Next, 10 μM 5-FU and 2 μg of pL3 were encapsulated in biocompatible nanoparticles (NPs) chemically conjugated with HA to achieve active tumor-targeting ability in CD44 overexpressing cancer cells. We showed the specific intracellular accumulation of NPs in cells and a sustained release for 5-FU and L3. Analysis of cytotoxicity and apoptotic induction potential of combined NPs clearly showed that the 5-FU plus L3 were more effective in inducing apoptosis than 5-FU or L3 alone. Furthermore, we show that the cancer-specific chemosensitizer effect of combined NPs may be dependent on L3 ability to affect 5-FU efflux by controlling P-gp (P-glycoprotein) expression. These results led us to propose a novel combined therapy with the use of 5-FU plus L3 in order to establish individualized therapy by examining L3 profiles in tumors to yield a better clinical outcomes.

## INTRODUCTION

In eukaryotes, ribosomal proteins (r-proteins) in addition to the role as components of translation machinery, exert a variety of extra-ribosomal functions [1–4].

These extraribosomal activities include DNA replication transcription and repair; RNA splicing and modification; cell growth and proliferation; regulation of apoptosis and cellular transformation. Some r-proteins take part to tumorigenesis by controlling oncogene and tumor suppressor expression [5].

Recent data show that some r-proteins can regulate the function of the tumor suppressor p53 [6]. Several r-proteins represent additional important component of p53 autoregulatory feedback loop and are shown to activate p53 and trigger cell cycle arrest and apoptosis [7, 8]. In addition, many r-proteins can themselves function as tumor suppressors.

In cancer cell proliferation the rate of ribosome production is essential to sustain cell growth and proliferation suggesting that cancer cells are more sensitive to nucleolar stress than normal cells. Thus, chemotherapeutic agents that selectively target ribosome biogenesis could be less toxic to normal and differentiated cells [9]. Many common anticancer drugs as 5-FU interfere with rRNA metabolism and ribosome biogenesis [10]. The resulting nucleolar stress causes the accumulation of free r-proteins which can exert their extraribosomal functions. A subset of these ribosome-free proteins activate different signaling pathways including ribosomal protein-Murine Double Minute 2-p53 pathway to mediate cell cycle arrest, apoptosis, differentiation and/or senescence [11]. Although these pathways are well studied, most cancer cells contain mutant p53 or no p53 at all and p53 independent pathways reveal a role of ribosomal proteins in regulation of nucleolar stress response [12].

5-FU is commonly used for the treatment of colon cancer. However, its antitumor activity is low (approximately 40%) mainly due to resistance. A great deal of effort has gone into identifying new contributing molecules that may enhance tumor response to 5-FU. The regulation of drug sensitivity by r-proteins has been already documented. To date, rpS3a is involved in regulation of cancer cell response to chemotherapy [13]. Mitochondrial rpL41 targets the activating transcription factor 4 (ATF4), a major regulator of tumor cell survival, for degradation contributing to sensitize tumor cells to chemotherapy [14].

Recently, we demonstrated that ribosome-free rpL3 (L3) represents a crucial player in cell response to nucleolar stress induced by 5-FU in colon and lung cancer cells devoid of p53 [15]. Apoptosis represents an importan event in the treatment of cancer. The success of a therapeutic strategy depends mainly on the capacity of the drug to induce apoptosis either by inhibiting antiapoptotic protein or by stimulating the expression of proapoptotic molecules. Recently, we have demonstrated that nucleolar stress upon 5-FU treatment leads to the activation of L3 as proapoptotic factor. In particular, L3 regulates CBS and p21 expression at transcriptional and post-translational levels leading to cell cycle arrest or apoptosis [16].

Our results prompted us to hypothize that the knowledge of L3 status in p53 null cancers may have a significant value in terms of the efficacy of chemotherapy based on 5-FU. In this study, we demonstrate that L3 expression in colon tumor tissues is downregulated; L3 mRNA decrease associated with malignance progression and tumor grade and was inversely proportional to the ratio Bcl-2/Bax; L3 overexpression in 5-FU treated colon cancer cells decreased clonogenic potency, cell migration and cell viability, and stimulated apoptotic cell death by inducing late apoptosis. The potential of this strategy for the treatment of colon cancer was next investigated by using novel polymeric nanoparticles based on a core of poly(lactic-co-glycolic) acid (PLGA) and a polymer shell of Hyaluronan (HA) and Polyethyleneimine (PEI) as platform to deliver the 5-FU and the proapoptotic protein L3 [17, 18]. Combined NP treated cells exhibited efficient cellular uptake and express high and prolonged levels of L3 protein and its target p21. Combined NP treatment resulted more effective in inducing apoptosis in cancer cells than 5-FU or L3 alone. In addition, we demonstrated that cytotoxic effect of combined NPs was due to the ability of L3 to destabilize MDR1 (Multi Drug Reactivity 1) mRNA and downregulate P-gp (P-glycoprotein) pump expression inhibiting, in this way, 5-FU efflux from treated cells.

Altogether these results led us to propose a novel combined therapy with the use of 5-FU along with pL3 in order to establish individualized therapy by examining L3 and p53 profiles in patient's tumors with the expectation to yield a better clinical outcomes.

## RESULTS

### L3 and L3 target gene expression profile in colon cancer clinical samples

**Figure 1:**
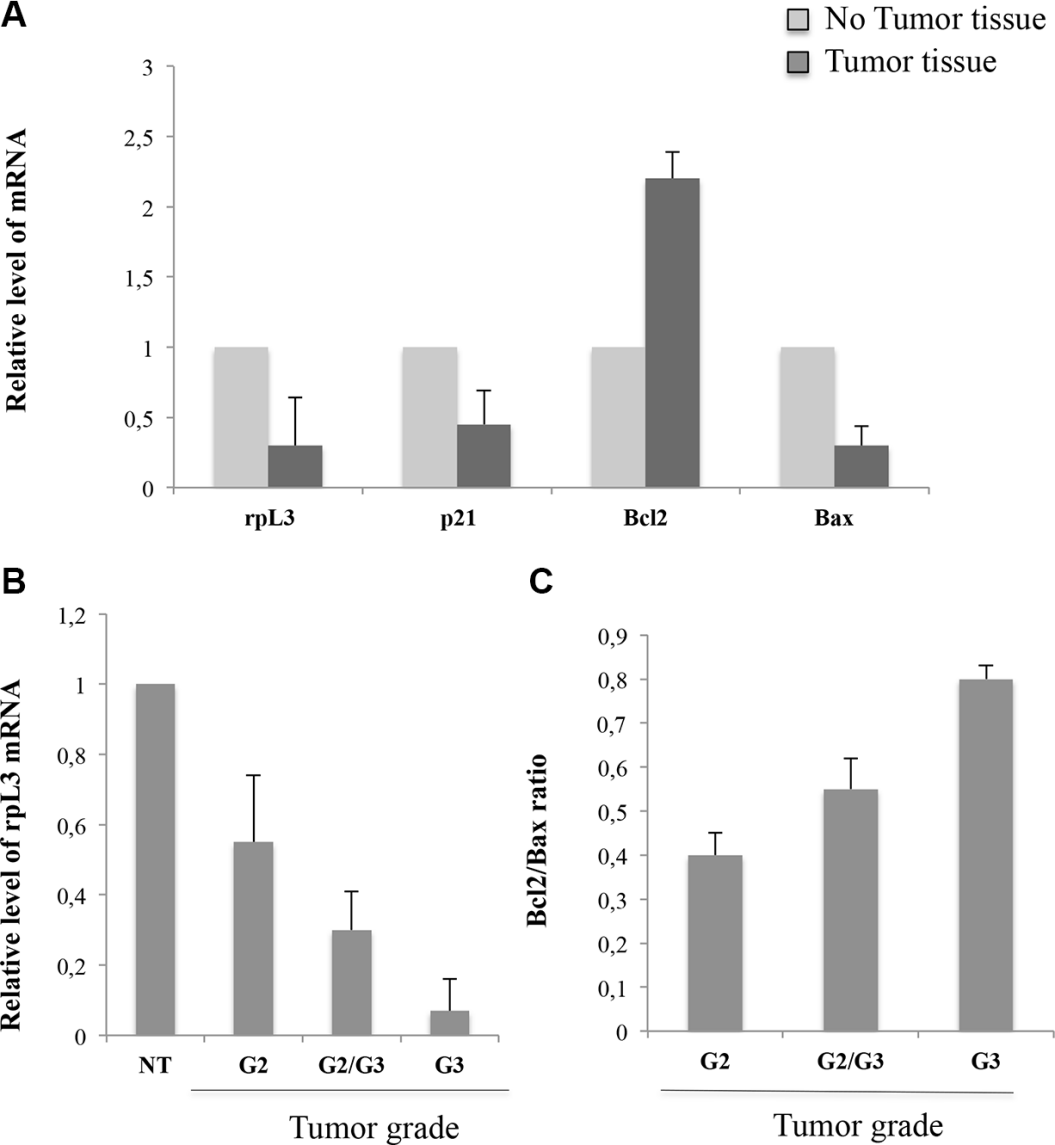
Expression profile of L3, p21, Bax and Bcl-2 in normal colon tumor tissues and colon adenocarcinoma qRT-PCR data showing (**A**) L3, p21, Bcl-2 and Bax mRNA levels in tumor tissues paired with the normal mucosa tissues set as 1. (**B**) L3 mRNA levels and (**C**) Bcl-2/Bax ratio among different groups of grade. NT: non tumor tissue. Results are shown as fold change (mean ± SEM) (*n* = 3) of normal mucosa tissues set as 1. Results illustrated in Figures 1–8, are representative of three independently performed experiments; error bars represent the standard deviation.

To evaluate L3 clinical significance in colon cancer, we employed quantitative real-time RT-PCR (qRT-PCR) to assess the expression of L3 and its target gene p21, Bcl-2 and Bax at the mRNA levels in 30 colon cancers and normal tissues. Comparison of human colon cancer specimens with patient-matched normal tissues revealed the down-regulation of L3 in tumors. As attended, we observed the down-regulation of p21 and Bax, and the up-regulation of Bcl-2 (Figure 1A). In addition, we observed that the downregulation of L3 expression along with increasing Bcl-2/Bax ratio correlated with increasing tumor grade (Figure 1B, 1C).

Table 1 summarizes demographic, pathological and clinical data of analyzed tissues.

**Table 1:** Demographic, pathological and clinical data of analyzed tissues

Case n°	Gender	Age	Differentiation	Grade	Stage
1	Female	72	Moderate	G2	pT2 pNO
2	Female	81	Moderate in situ	G2/3	pTis pNO
3	Female	38	Poor	G2	pT2 pN1a
4	Female	58	Moderate, Ulcerated	G2	pT2 pNO
5	Female	66	Poor	G2/3	ypT2 ypN1b
6	Male	70	Moderate	G2	pT2 pNO
7	Male	67	Poor, Mucinous	G3	pT3 pNO
8	Male	70	Poor, Ulcerated	G3	pT4b pN1b
9	Male	56	Moderate	G2	pT2 pNO
10	Male	48	Moderate	G2	pT2 pNO
11	Male	51	Moderate, Mucinous	G2	pT2 pNO
12	Male	78	Moderate, Ulcerated	G2	pT3 pN1a
13	Male	72	Poor, Mucinous	G2/3	pT3 pN2a
14	Female	82	Moderate, Mucinous	G2	pT2 pNO
15	Male	73	Moderate, Ulcerated	G2	pT2 pN1a
16	Male	67	Moderate	G2	pT2 pNO
17	Male	47	Poor	G3	pT3 pN2b
18	Male	62	Moderate, Ulcerated	G2	pT3 pN2b
19	Male	85	Moderate, Mucinous	G2	pT2 pNO
20	Male	57	Moderate	G2	pT3 pNO
21	Female	78	Moderate	G2	pT2 pNO
22	Male	78	Moderate	G2	pT2 pNO

### L3 enhances 5-FU mediated inhibition of cell viability, clonogenicity and migration

**Figure 2:**
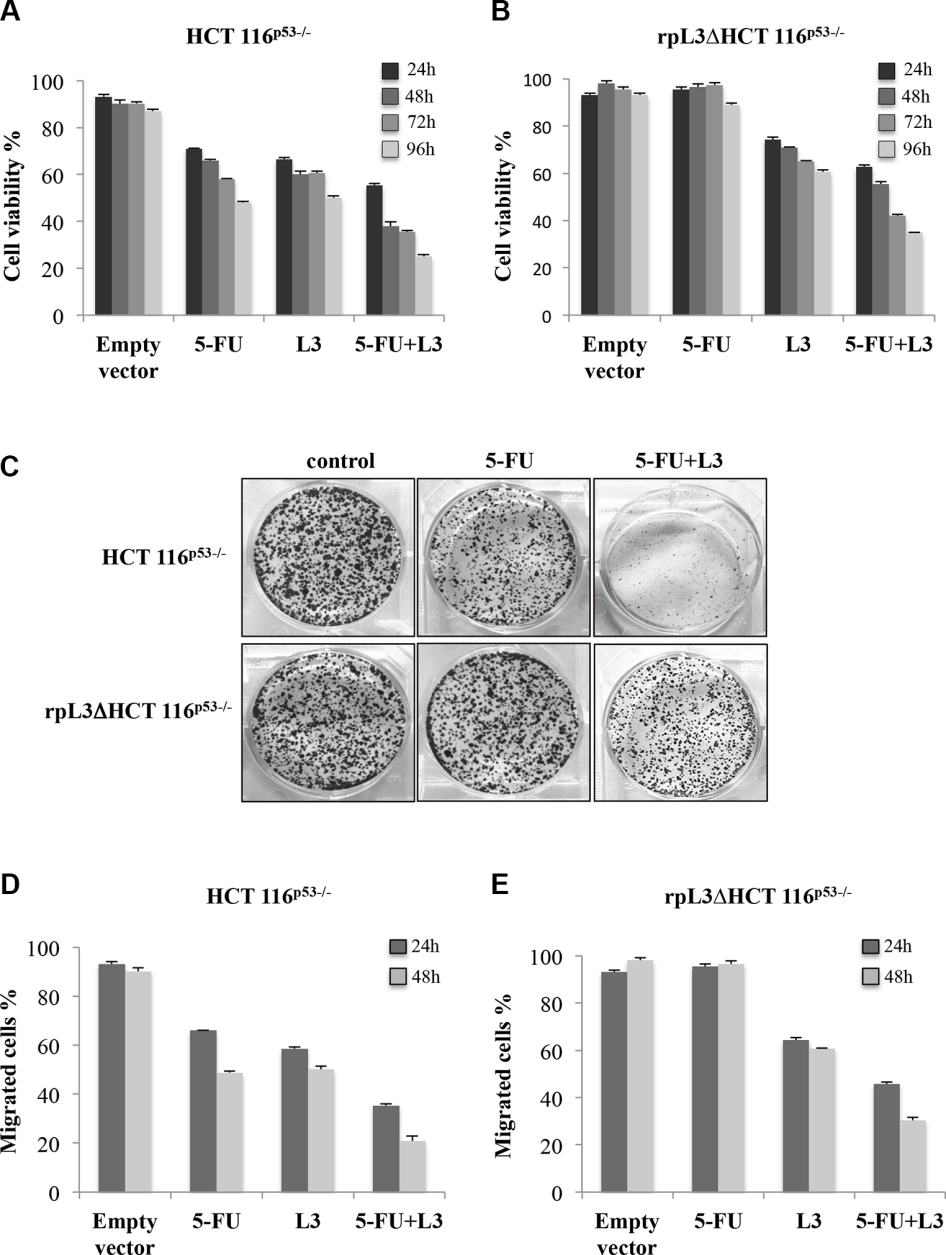
Role of L3 on cell viability, cell proliferation and migration upon 5-FU treatment (**A**) HCT 116^p53−/−^ and (**B**) rpL3ΔHCT 116^p53−/−^ cells were transiently transfected with pL3 and treated with 10 μM 5-FU for 24 h, 48 h, 72 h and 96 h or untreated. Then, cell viability was evaluated using MTT assay. The cell viability from untreated cells was set to 100%. Results are presented as percentage (mean ± SEM) (*n* = 3) of the control cells. (**C**) Representative image of clonogenic analysis for cell proliferation in HCT 116^p53−/−^and rpL3ΔHCT 116^p53−/−^ cells upon L3 overexpression and 5-FU treatment for 48 h. After 7 days, colonies were stained with methylene blue, photographed and counted. (**D**) HCT 116^p53−/−^ and (E) rpL3ΔHCT 116^p53−/−^cells were transiently transfected with pL3 and treated with 10 μM 5-FU for 24 h and 48 h or untreated. Then migration of cells was examined using Boyden chamber. Cell migration of untreated cells was set to 100%. Results are presented as percentage (mean ± SEM) (*n* = 3) of the control cells.

The analysis of L3 expression profile in tumors prompeted us to investigate whether the up-regulation of L3 may potentiate the cytotoxic activity of 5-FU. We have previously demonstrated that L3 may induce G1/S cell cycle arrest or apoptosis depending on its concentration inside the cell [19]. With the aim of examining the combined effect of L3 and 5-FU on cell viability, HCT 116^p53−/−^ cells and rpL3ΔHCT 116^p53−/−^ cells, a cell line stably depleted of L3 [20], were transiently transfected with a pro-apoptotic concentration of a plasmid encoding L3-GFP fusion protein (pL3, 2 μg) and treated or not with a subtoxic dose of 5-FU (10 μM). 24 h, 48 h, 72 h and 96 h later, cell viability was estimated by MTT assay. As expected, the percentage of cell viability decreased after 5-FU treatment or pL3 transfection compared to untreated HCT 116^p53−/−^ cells, control. Of note, in cells transfected with pL3 and treated with 5-FU we found that the cell viability was strongly reduced compared with cells exposed to 5-FU or transfected with pL3 (Figure 2A). In rpL3ΔHCT 116^p53−/−^ cells, L3 silencing completely abolished the cytotoxicity of 5-FU treatment (Figure 2B). Nevertheless, this effect was directly rescued by pL3 transfection (Figure 2B) confirming the crucial role of this protein in 5-FU cell response [15]. Of note, transfection in these cells of pL3 together with 5-FU treatment increased the percentage of cell cytotoxicity as compared with that of cells transfected with L3 or treated with 5-FU (Figure 2B).

We further analysed the influence of L3 and 5-FU treatment on cell proliferation by performing a clonogenic assay. To this aim, HCT 116^p53−/−^ and rpL3ΔHCT 116^p53−/−^ cells were pre-treated with 10 μM 5-FU for 48 h, then transiently transfected with 2 μg of pL3. Figure 2C shows a reduction of colony number of HCT 116^p53−/−^ cells upon exposure to 5-FU confirming the ability of the drug to inhibit clonogenicity. The capacity of rpL3ΔHCT 116^p53−/−^ cells to produce colonies upon 5-FU treatment was comparable to that of untreated cells confirming that the loss of L3 plays an important role in the inhibition of cell proliferation upon exposure to 5-FU. It is noteworthy that in both cell lines pL3 transfection and 5-FU treatment resulted in a further reduction of clonogenicity confirming the ability of L3 to improve the cytotoxic activity of 5-FU. The effect of rpL3 on cell viability and clonogenicity was confirmed in HT29 cells, an other human colon cancer cell line non harboring p53 (Supplementary Figure S1).

Furthermore, we investigated the role of L3 overexpression alone or in combination with 5-FU on cell migration. To this purpose, HCT 116^p53−/−^ cells were transiently transfected with pL3 and treated with 10 μM 5-FU for 24 h and 48 h. Then, cell migration was analyzed by using Boyden chamber migration assay. As shown in Figure 2D, the migration ability of 5-FU treated HCT 116^p53−/−^ cells was reduced of about 40% and 50% at 24 h and 48 h, respectively, as compared with untreated cells set as 100%, control. When rpL3 was overexpressed, the migration ability of 5-FU treated HCT 116^p53−/−^ cells was further reduced (60% and 80% at 24 h and 48 h, respectively, vs untreated cells set as 100%, control) demonstrating that L3 overexpression was able to improve 5-FU mediated inhibition of cell motility. Additionally, we demonstrated that inhibition of cells migration was specifically mediated by L3. For this purpose, analogous experiments were performed in rpL3ΔHCT 116^p53−/−^ cells. In this cell line, 5-FU treatment failed to inhibit cell migration; of note, the transfection of pL3 together with 5-FU treatment were able to rescue 5-FU activity (Figure 2E and Supplementary Figure S2). Quantification of migrated cell number indicated that the overexpression of L3 along with 5-FU treatment reduced cell migration of about 50% and 70% at 24 h and 48 h, respectively.

### L3 enhances 5-FU mediated apoptotic response of HCT 116^p53−/−^ cells

**Figure 3:**
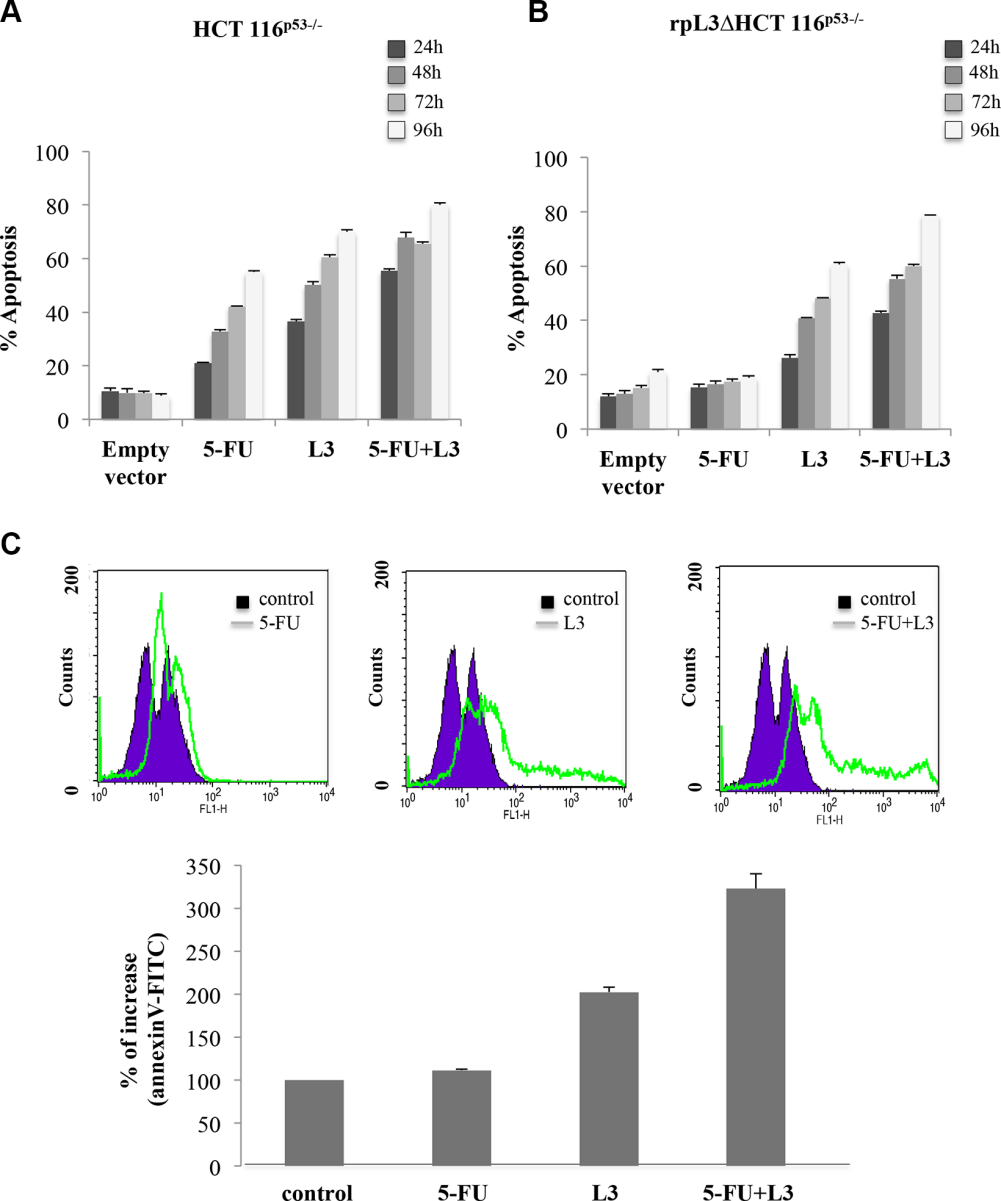
Role of rpL3 on apoptosis upon 5-FU treatment (**A**) HCT 116^p53−/−^ and (**B**) rpL3ΔHCT 116^p53−/−^ cells were transiently transfected with pL3 and treated with 10 μM 5-FU for 48 h or untreated. Then cells were analyzed for mitochondrial membrane potential by TMRE staining. Fluorescence was measured by flow cytometry. Results are presented as percentage of the control cells set as 100%. Results are presented as percentage (mean ± SEM) (*n* = 3) of the control cells. (**C**) HCT 116^p53−/−^ cells were transiently transfected with pL3 and treated with 10 μM 5-FU for 48 h. Then, cell death was assessed by FACS analysis of Annexin V staining. Quantitative data are reported.

To determine whether L3 decreased cell survival by inducing apoptosis, we analyzed the reduction of mitochondrial inner membrane potential (ΔΨm) by tetramethyl-rhodamine ethyl ester (TMRE) staining, hallmarks of mitochondrial apoptosis. To this aim, HCT 116^p53−/−^ and rpL3ΔHCT 116^p53−/−^ cells were treated with 10 μM 5-FU and transfected with 2 μg of pL3. Figure 3A and 3B show that the percentage of apoptosis increased in cells treated with the combination of pL3 plus 5-FU compared to that of cells treated with 5-FU or pL3 alone in each cell line.

To ascertain the above observation, Annexin V analysis was performed. HCT 116^p53−/−^ cells were transfected with 2 μg of pL3, treated with 5-FU for 48 h and then evaluated with Annexin V-FITC. As shown in Figure 3C, the percentage of cells Annexin V positive increased after 5-FU treatment and pL3 transfection compared to cells untreated or treated with 5-FU or pL3 alone.

**Figure 4:**
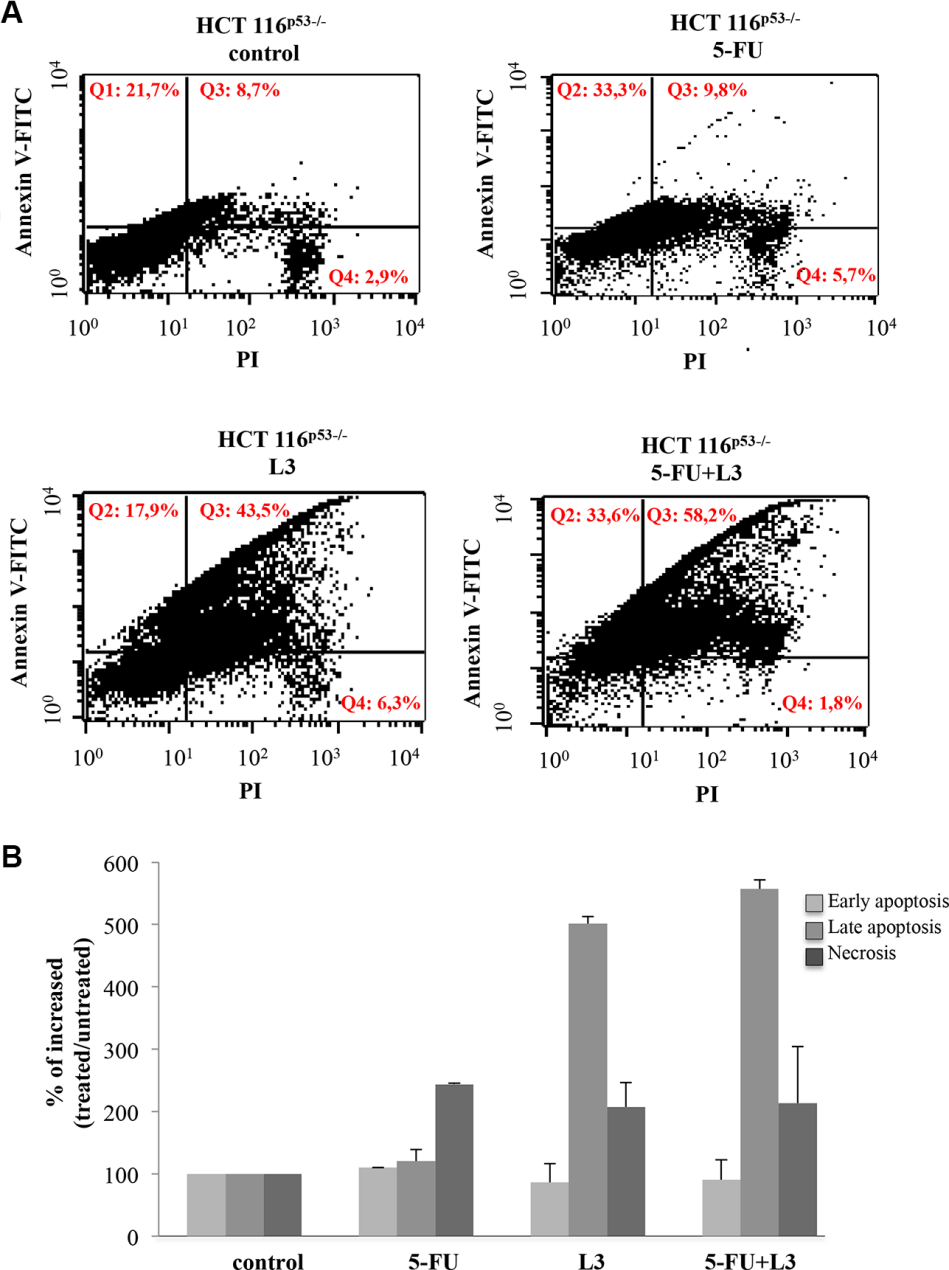
Analysis of apoptosis status upon combined treatment 5-FU+L3 HCT 116^p53−/−^ cells were transiently transfected with pL3 and treated with 10 μM 5-FU for 48 h. Then, cell death was assessed by FACS analysis of Annexin V and PI staining. (**A**) Representative dot plots and (**B**) quantitative data are reported.

These results were further confirmed by Annexin V-FITC/PI dual staining. Specifically, we found that pL3 transfection significantly increased the percentage of late apoptotic cells (Annexin V^+^ and PI^+^) from 10% in the untreatd cells to 50% in pL3-transfected cells (Figure 4). Furthermore, in agreement with cell growth inhibition, the combination treatment of pL3 plus 5-FU was able to reduce the number of necrotic cells induced by 5-FU treatment alone and to induce a higher number of Annexin V^+^ and PI^+^ cells, i.e. apoptotic cells, than the treatment with 5-FU or pL3 alone (Figure 4). All together these results strongly suggest that the ectopic expression of L3 allowed a more potent cytotoxic effect of 5-FU on colon cancer cells. On this basis, delivery of pL3/5-FU combination in HCT 116^p53−/−^ cells through biodegradable NPs was attempted.

### Delivery of 5-FU and pL3 to HCT 116^p53−/−^ cells with targeted nanoconstructs

**Figure 5:**
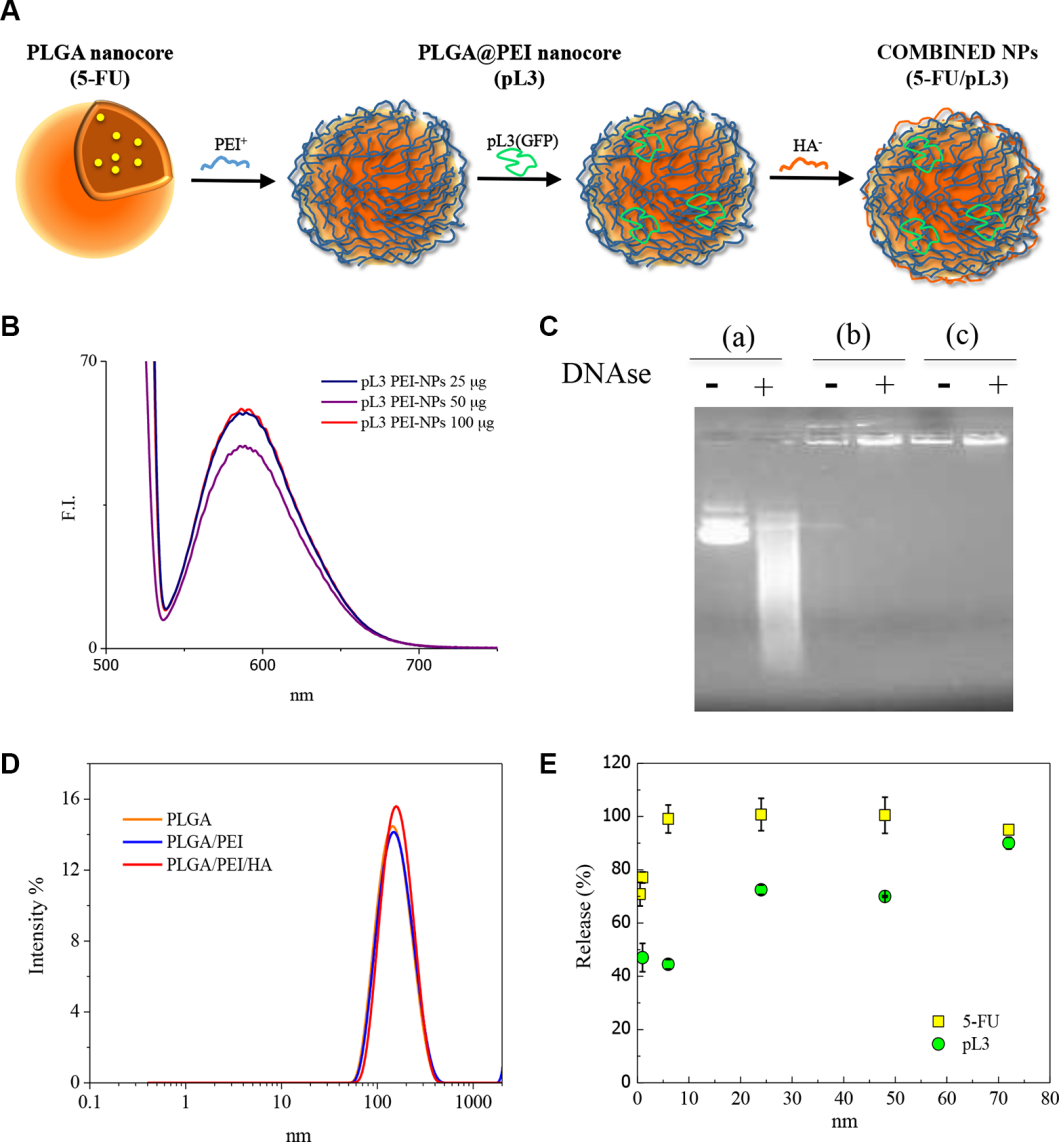
Structure and characterization of NPs delivering 5-FU+pL3 (**A**) Sketched representation of NPs delivering 5-FU+pL3. (**B**) Emission spectra (excitation = 530 nm) of ethidium bromide in the presence of pL3-loaded PLGA@PEI (0–100 μg/mL). (**C**) Gel retardation assay before and after sample treatment with DNAse: free pL3 (a), pL3-loaded PLGA@PEI (b), pL3-loaded HA-coated PLGA@PEI (c), (pL3 was 2 μg/mL). (**D**) Size distribution of the sample during the layering procedure. (**E**) Release profile of 5-FU and pL3 from combined NPs in DMEM FBS^+^.

Multistep preparation of combined NPs (Figure 5A) comprised: i) the preparation of a negatively-charged PLGA nanocore entrapping 5-FU; ii) the adsorption of a cationic PEI layer (PLGA@PEI nanocore); iii) the adsorption of pL3 onto PLGA@PEI nanocore; iv) the deposition of an anionic decorating layer of HA to give final combined NPs. The loading capability of the separate drugs in NPs was preliminary evaluated (Supplementary Table S1). Amounts of 5-FU up to 0.9 mg could be loaded in 100 mg of PLGA nanocores with size below 200 nm. PLGA nanocores were negative and then coated with the polycation PEI (around 80 mg/mg PLGA nanocore) as previously reported [18]. To load the plasmid, different amounts of pL3 were incubated with PLGA@PEI nanocore dispersed in water and effectively adsorbed on the surface (Supplementary Table S2). Then, pL3 adsorption, mean hydrodynamic diameter and PI did not change up to a concentration of 10.4 mg/mL where a macroscopic aggregation occurred (Supplementary Table S2). In a separate experiment aimed at evaluating the association of pL3 to PLGA@PEI nanocores, EtBr fluorescence emission due to pL3 intercalation was monitored. It was found that the emission of EtBr/pL3complex progressively decreased at increasing concentrations of PLGA@PEI nanocores, due to hampered intercalation of EtBr to pL3 adsorbed onto NPs (Figure 5B). Upon pL3 adsorption, NP zeta potential was still positive (Supplementary Table S2) due to the presence of free cationic amine groups on PEI on the surface, thus allowing the final decoration with a HA layer. After addition of HA, zeta potential was reversed from positive to negative values due to HA interaction with PEI.

To evaluate the effective association and protection of plasmid when loaded onto PEI-coated PLGA core, without and with the final HA layer (cationic and anionic, respectively), a gel retardation assay was performed. The run of pL3 was retarded in case of PLGA@PEI nanocore, without and with HA coating (Figure 5C, lines b and c, respectively), as compared to free pL3 (Figure 5C, line a). After treatment of pL3-loaded NPs with DNAse, no change was observed in the runs of NPs (Figure 5C, line c) while free pL3 was readily degraded (Figure 5C, line a). From the comparison of results for PLGA@PEI nanocore without and with HA coating, it was evident that the adsorption of HA did not alter the association extent of pL3 to NPs.

On this basis, we prepared combined NPs with different 5-FU/pL3 reciprocal amounts and evaluated their properties (Table 2 and Supplementary Table S2). Final NPs decorated with a HA layer were below 200 nm with a negative zeta potential and could be freeze dried after addition of trehalose. NPs could be redispersed in water or DMEM with FBS 10% giving stable colloidal dispersion with a limited increase of size and without any macroscopic aggregation over time in agreement with our previous results (data not shown) [18]. Thus, through this NP design we could easily regulate the reciprocal amount of both 5-FU and pL3 by adjusting the experimental conditions and without altering the overall properties of NPs relevant for biological applications.

**Table 2:** Properties of combined HA-coated NPs loaded with 5-FU and pL3

Formulation code^a^	5-FU Act. Load. (mg/100 mg)	pL3 Act. Load. (mg/100 mg)	Hydrodynamic diameter (nm ± SD)	P.I.	Zeta potential (mV)
**5-FU_L_/pL3_L_NPs**	0.20 ± 0. 05	0.15 ± 0.1	160 ± 5	0.10	−26
**5-FU_M_/pL3_L_NPs**	0.40 ± 0.02	0.15 ± 0.1	165 ± 10	0.13	−25
**5-FU_H_/pL3_L_NPs**	0.88 ± 0.1	0.15 ± 0.1	165 ± 18	0.13	−25
**5-FU_H_/pL3_H_NPs**	0.88 ± 0.1	0.36 ± 0.1	181 ± 8	0.20	−22

NP acronyms used are composed by character strings indicating: bioactive cargo (5-FU and/or pL3) and its theoretical loading which is Low (L), medium (M) and high (H). In the case of 5-FU, L, M and H theoretical loading was 2, 5 and 10 mg per 100 mg of polymer, respectively. In the case of pL3, L and H theoretical loading was 2.6 and 5.2 μg per 0.5 mg of PEI-coated PLGA NPs.

Release profile of 5-FU and pL3 from NPs was assessed indirectly from the amount remaining in the NPs upon incubation in DMEM FBS+. An illustrative curve for the results (Figure 5D) suggest that the release was around 50% within the first 3 h while it was around 80% after 24 h. The results are in agreement with Su et al. who showed a similar behavior for PLGA/PEI-NP [21]. 5-FU release was sustained and around 100% of total drug content was released after 6 h.

### *In vitro* activity of 5-FU NP_S_ and pL3_H_NPs in HCT 116^p53−/−^ cells

**Figure 6:**
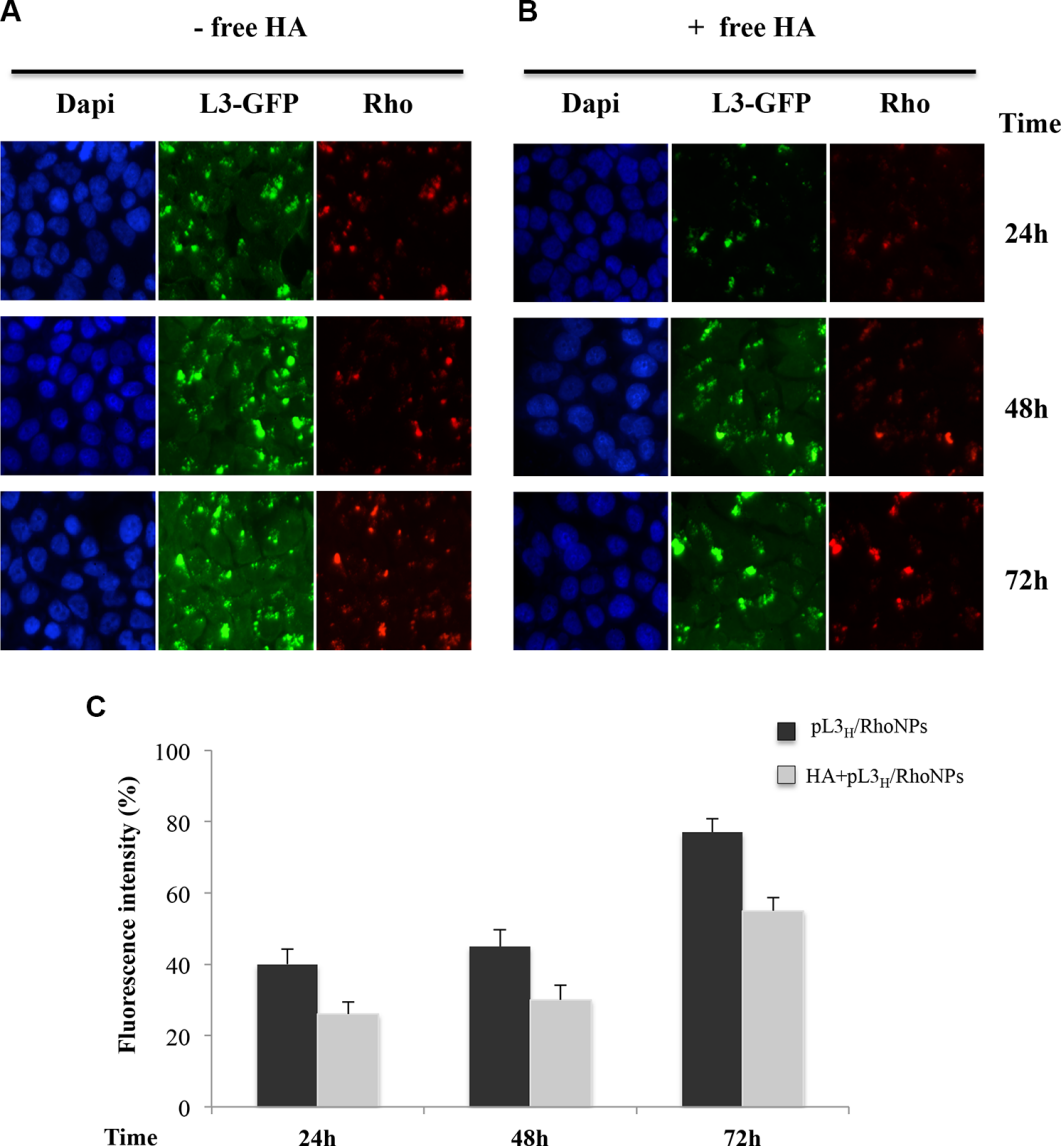
Cellular uptake of pL3_H_/RhoNPs Representative fluorescent miscroscopy images of HCT 116^p53−/−^ cells treated with pL3_H_/RhoNPs for 24 h, 48 h and 72 h. Dapi was used as a nuclear stain (shown in blue); L3-GFP and NP dependent fluorescence (Rho) are shown in green and red, respectively. The CD44 receptor of HCT 116^p53−/−^ cells was blocked with free HA 1 h before treatment with nanoparticles. Quantification of fluorescence intensity is shown. Results are presented as percentage (mean ± SEM) (*n* = 3) of the control cells set as 100%.

The cytotoxicity of 5-FU NPs in HCT 116 ^p53−/−^ cells was preliminary tested. To this aim, HCT 116 ^p53−/−^ cells were treated with a wide range of concentrations (0.05–100 μM) of 5-FU NPs and *in vitro* cytotoxicity was evaluated after 72 h and 96 h of exposure by using MTT assay and compared to that of free 5-FU. As shown in Supplementary Figure S3, a significant decrease in the IC_50_ was observed in cells treated with 5-FU NPs either at 72 h or 96 h (IC_50_ 5 μM and 1 μM, respectively) compared to cells treated with free 5-FU (IC_50_ 10 μM and 5 μM, respectively). These results suggest that NPs enhanced the cytotoxic activity of 5-FU in HCT 116 ^p53−/−^ cells. To investigate CD44-mediated delivery of NPs, CD44 receptor-overexpressing HCT116 ^p53−/−^ cells were incubated with fluorescent NPs labeled with Rhodamine (pL3_H_/RhoNPs) from 24 h to 72 h and observed under the microscope. As shown in Figure 6A and 6B, the incubation of cells with pL3_H_/RhoNPs lead to an efficient and prolonged expression (until 72 h) of L3-GFP in HCT 116 ^p53−/−^ cells. Moreover, to better characterize the ability of pL3_H_NPs to allow the delivery into the cells of L3, we compared their behavior with that of a commercially available DNA-transfecting agent as Lipofectamine 3000 (Lipo) by using GFP-fluorescence analysis (Supplementary Figure S4). Comparing results in Figure 6, panel A and Supplementary Figure S4 both NP and Lipo mediated transfection lead to an expression of L3-GFP in tumor cells. Of note, NPs drastically enhanced transfection efficiency. In fact, cells transfected with pL3_H_/RhoNPs showed a sustained and a more efficient expression of L3-GFP as a function of time. In fact, the results demonstrated that there was a significant increase in fluorescence intensity at 72 h time point suggesting the slow release of DNA from NPs localized inside the cells. In contrast, Lipo failed to mantain high expression levels of L3-GFP over time (Supplementary Figure S4). These data suggest that events downstream of NP internalization as intracellular trafficking, uptake and processing may be additional determinants of NP-mediated L3 expression efficiency.

Furthermore, fluorescence intensity was significantly decreased when the CD44 receptor was blocked by pretreatment with free HA (Figure 6B, 6C) indicating that HA-coated NPs can target tumor cells through receptor-mediated endocytosis using the CD44 receptor.

### Targeted delivery of L3 loaded nanoparticles enhances 5-FU sensitivity of p53 null colon cancer cells

**Figure 7:**
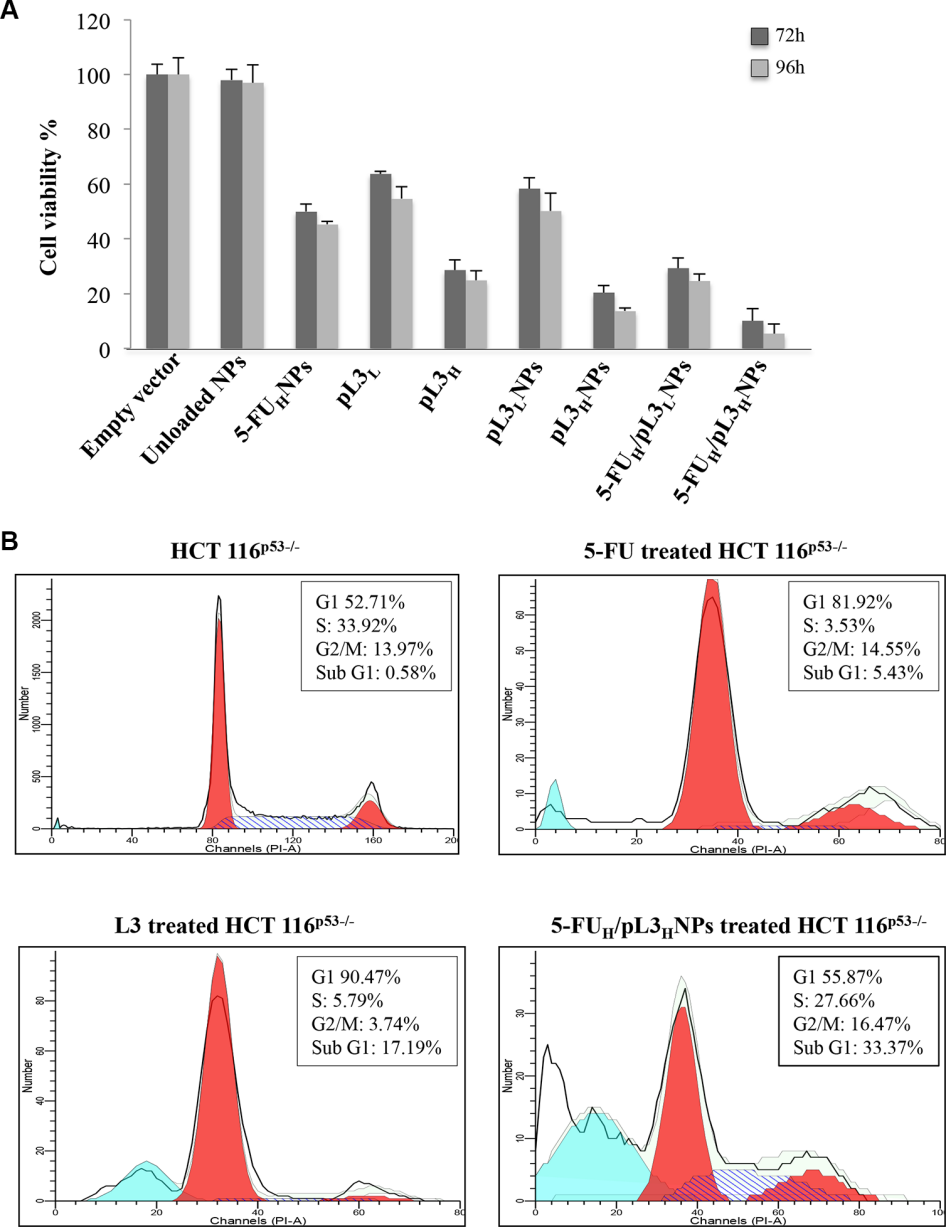
Effect of 5-FU_H_/pL3_H_NPs on cell viability and cell cycle in HCT 116^p53−/−^ cells (**A**) HCT 116^p53−/−^ were treated with 2 μg of empty vector, unloaded NPs, 10 μM 5-FU loaded onto NPs (5-FU_H_NPs), 1 mg or 2 mg of free pL3 (pL3_L_ or pL3_H,_ respectively), 1 μg or 2 μg of pL3 loaded onto NPs (pL3_L_NPs or pL3_H_NPs, respectively), 10 μM 5-FU plus 1 μg or 2 μg of pL3 loaded onto NPs (5-FU_H_/pL3_L_NPs and 5-FU_H_/pL3_H_NPs, respectively) for 72 h and 96 h. After incubation, cell viability was evaluated using the MTT assay. The cell viability from untreated cells was set to 100%, control. Results are presented as percentage (mean ± SEM) (*n* = 3) of the control cells. (**B**) HCT 116^p53−/−^ cells were treated with pL3_H_, 5-FU or 5-FU_H_/pL3_H_NPs for 48 h. Then, cells were stained with PI and analysed using FACS. Peaks representing histograms of cell numbers and percentages in G1, S, and G2/M phases are shown.

To investigate the cytotoxic activity of the 5-FU_H_/pL3_H_NPs as potential targeted tumor therapy, the inhibitory effects of different combinations of L3 and 5-FU were compared with single treatments in HCT 116^p53−/−^ cells by MTT assay. We have previously demonstrated that L3 may induce G1/S cell cycle arrest or apoptosis depending on its concentration inside the cell [19]. Thus, in combination treatment, 10 μM 5-FU was applied along with two different amounts of pL3, 1 μg e 2 μg corresponding to the cytostatic and the cytotoxic dose, respectively [19]. The results of the cell viability with different treatments obtained after 72 h and 96 h of incubation were shown in Figure 7A. Unloaded NPs exhibited no toxicity at tested concentrations and time points. As attended, the treatment of cells with 1 μ of pL3, free or loaded onto NPs (pL3_L_ or pL3_L_NPs, respectively) exhibited about 44% and 55% of cell viability inhibition at 72 h and 96 h, respectively, vs. untreated cells set as 100%, control. At cytotoxic dose (2 μg), free pL3 (pL3_H_) showed higher cytotoxic effect (71% and 76% at 72 h and 96 h, respectively, vs. control). A further increase of about 10% of cytotoxic activity was observed when the same amount of pL3 was loaded onto NPs (pL3_L_NPs). Combination treatment of 10 μM 5-FU with 1 mg or 2 mg of pL3 (5-FU_H_/pL3_L_NPs or 5-FU_H_/pL3_H_NPs, respectively) generated significantly higher antiproliferation effects compared to either agent alone (pL3 or 5-FU). Of note, the treatment of cells with 5-FU_H_/pL3_H_NPs showed much lower cell viability compared to that observed by incubating cells with 5-FU_H_/pL3_L_NPs indicating a strong potential for the combination treatment carrying higher experimented amount of pL3. In fact, FU_H_/pL3_H_NP treated cells enhanced the cytotoxicity of 5-FU in HCT 116^p53−/−^ cells by about 40% compared to 5-FU alone indicating that this combination of L3 with 5-FU might be promising.

With the aim of further examinin the combined effect of L3 and 5-FU on cell proliferation, alterations in the were analyzed. To this aim, cells were treated with 5-FU_H_/pL3_H_NP and 48 h later cell cycle distribution was monitored by flow cytometry. As shown in Figure 7B 5-FU treated cells resulted in an enlarged proportion of cells in G1/S phase arrest. Of note, upon the treatment of cells with 5-FU_H_/pL3_H_NP we observed only a mild effect on the cell cycle, and a a significant sub-G1 population indicative of apoptosis (about 30% vs. 5-FU treated cells 5%).

All together these results demonstrate the potential enhancement of 5-FU based cancer therapy by L3.

### P-gp is reduced following L3 overexpression in p53 null colon cancer cells

**Figure 8:**
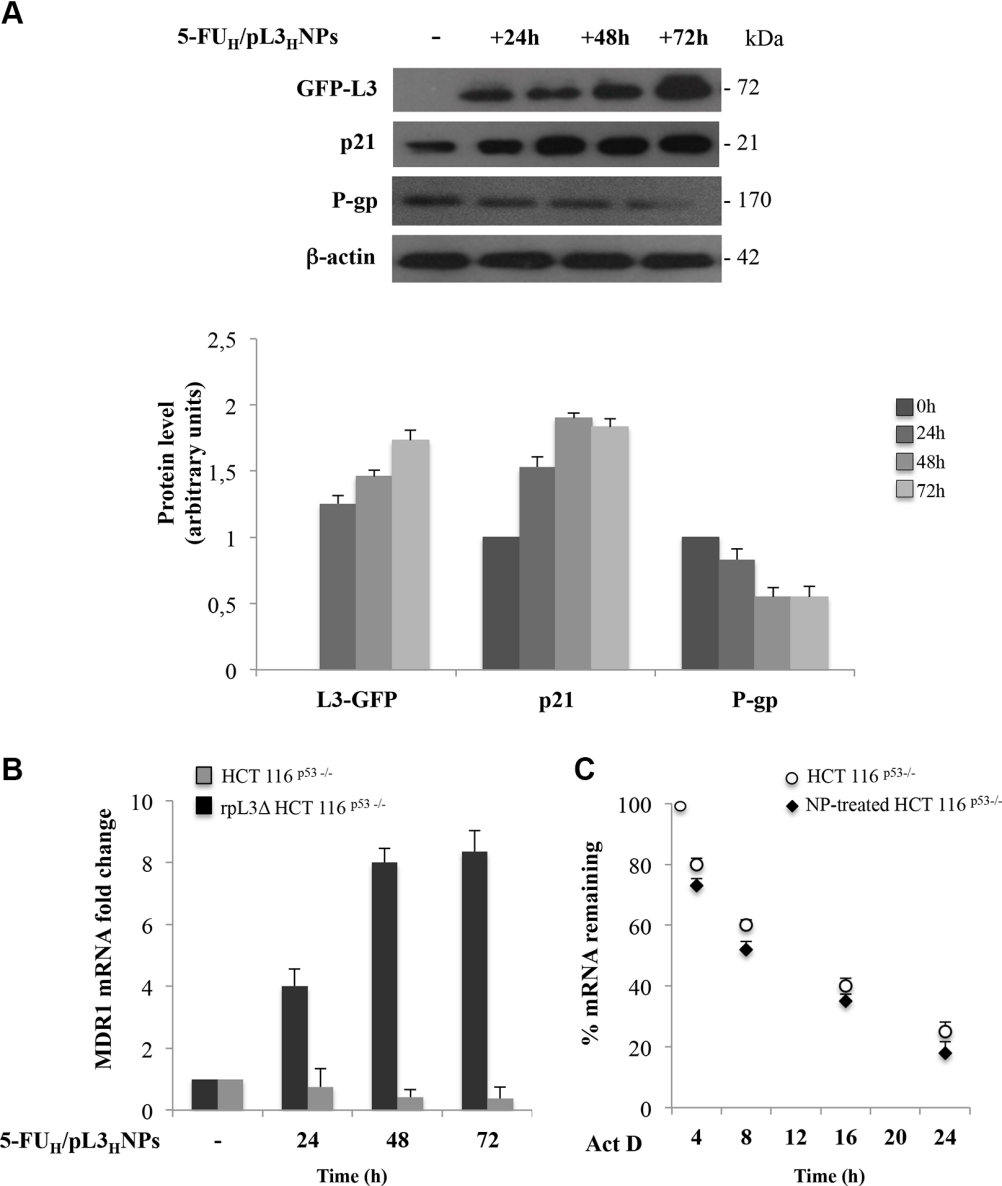
5-FU_H_/pL3_H_NP treatment negatively regulates P-gp expression and MDR1 mRNA stability (**A**) Representative western blot analysis showing time-dependent changes in L3-GFP, p21 and P-gp protein expression in HCT 116^p53−/−^ cells after treatment with 5-FU_H_/pL3_H_NPs for 24 h, 48 h and 72 h. Densitometric quantification is shown. (**B**) HCT 116^p53−/−^ and rpL3ΔHCT 116^p53−/−^ cells were treated with 5-FU_H_/pL3_H_NPs for 24 h, 48 h and 72 h. Then, mRNA expression of MDR1 was detected by qRT-PCR. Quantification of signals is shown. (**C**) HCT 116^p53−/−^ cells were treated with 5-FU_H_/pL3_H_NPs for 48 h. Then, 5 μg/μl Act D was added to the cells for 24 h. At the indicated time points (4 h, 8 h, 16 h and 24 h), total RNA was isolated and the mRNA levels of MDR1 and β-actin were determined by real-time PCR. The relative amount of MDR1 mRNA without Act D treatment was set to 100% and the percentage of MDR1 mRNA treated with Act D was calculated accordingly. Quantification of signals is shown.

In order to verify whether L3 protein over-expressed upon 5-FU_H_/pL3_H_NPs treatment was functional in the induction of its target gene p21, protein extracts from HCT 116 ^p53−/−^ cells treated with 5-FU_H_/pL3_H_NPs for 24 h, 48 h and 72 h were analyzed by western blotting using antibodies against L3, p21 and β-actin as control. Figure 8A shows that in cells exposed to 5-FU_H_/pL3_H_NPs the over-expression of L3 associated to a time dependent increase of p21 protein amount. In addition, to explore alterations in P-gp expression, an ATP-dependent membrane transport protein highly expressed in 5-FU resistant cancer cells [22], the expression profile of P-gp was assessed in these conditions. We surprisingly found that P-gp protein level decreased in a time dependent manner upon 5-FU_H_/pL3_H_NP treatment (Figure 8A). The level of MDR1 mRNA expression is an important determinant of P-gp expression levels. Thus, we detected mRNA level of MDR1 in HCT 116^p53−/−^ cells after NP treatment. To this aim, cells were incubated with 5-FU_H_/pL3_H_NPs for 24 h, 48 h, and 72 h. Then, cells were lysated and total RNA analyzed by qRT-PCR with primers specific for MDR1 and β-actin as control. The results showed that 5-FU_H_/pL3_H_NP treatment significantly decreased the amount of MDR1 transcript (Figure 8B). To evaluate the importance of L3 in the alteration of MDR1 mRNA level, analogous experiments were performed in rpL3ΔHCT 116^p53−/−^.cells. The results demonstrated that the silencing of L3 was associated to a strong increase of MDR1 mRNA level (Figure 8B). Next, we investigated whether 5-FU_H_/pL3_H_NP treatment could affect MDR1 mRNA half-life. To this aim, cells were incubated with combined NPs for 48 h after which the transcription inhibitor Actinomycin D (Act-D) was added for the following 24 h to inhibit nascent RNA synthesis. Total RNA was obtained from the samples at the indicated times (4 h, 8 h, 16 h and 24 h) and MDR1 and β-actin mRNA levels were analyzed by qRT-PCR. The results show that the half-life of MDR1 mRNA was decreased from approximately 10 h in normal HCT 116^p53−/−^ cells to less than 8 h in NP-treated cells (Figure 8C). These results indicate that the down-regulation of MDR1 mRNA levels following NP treatment could be due in part to a decrease in mRNA stability.

## DISCUSSION

Colon cancer is one of the leading causes of cancer-related death worldwide [23]. Although 5-FU is an old anticancer drug, it is still used as first line therapy in the treatment of colon cancer [24]. However, the therapeutic application of 5-FU is limited by its poor specificity, low bioavailability and serious drug-related side effects. In particular, the main cause of failure in colon cancer therapy is represented by the insurgence of resistance to 5-FU in the course of treatment [24]. To overcome these limitations new therapeutic strategies and/or new adjuvant drugs need to be explored. Colon cancer has been associated with alterations in apoptosis regulation [25] and, in particular, the inhibition of apoptosis is a crucial element in carcinogenesis of colorectal cancer as other human malignancies [26]. Unlike necrosis, apoptosis is an important cell death mechanism that does not trigger an inflammatory response leading to collateral destruction of normal cells in the surrounding microenvironment [27]. Cancer cells, however, exhibit resistance to apoptosis in order to sustain their uncontrolled proliferation and, therefore, any apoptosis-modulating compound is desirable as a plausible chemotherapeutic agent against cancer [28]. In particular, the restoration of signaling pathways involved in apoptosis induction, based on targeted multitherapy, may result in improved response to 5-FU treatment, which translates into decreased resistance to cancer therapy. In the present study, we demonstrate the potentiation of the cytotoxic effect of 5-FU on human colon cancer cells by proapoptotic L3 and aimed to a combined therapy with the use of 5-FU along with cDNA encoding L3 in order to establish individualized combined therapy by examining L3 and p53 profiles in patient's tumors with the expectation to yield a better clinical outcomes.

L3 is a component of the large subunit of cytoplasmic ribosomes. However, it functions not only within the ribosome participating in translation but also as an extraribosomal player involved in a number of cellular events. L3 autoregulates its own expression through the association of alternative splicing and nonsense-mediated mRNA decay [1]. Identified protein factors involved in promoting or inhibiting the alternative splicing of L3 pre-mRNA include hnRNP H1, Sp1 and NPM [29]. The action of ribosome free L3 has been intensively studied by us and the results highlighted its favourable anticancer potential over different conventionally used chemotherapeutic drugs as L-OHP, ActD and 5-FU [15, 20]. In particular, our studies have shown that L3 regulates transcription of many genes in response to 5-FU induced nucleolar stress [19, 20]. A key extraribosomal role of L3 is to block cell cycle progression and/or to induce apoptosis in response to drug-induced nucleolar stress. L3 can induce G1 arrest through the activation of the cyclin-dependent kinase inhibitor p21 transcription and apoptosis by molecular pathways involving p21 and CBS [16]. Analysis of L3 mRNA expression profile demonstrates that L3 is implicated in colon cancer (Figure 1A). In particular, the expression of L3 in colon cancer patients was associated to an advanced tumor stage (Figure 1B). In fact, we found that L3 mRNA amount decreased with malignant progression and the intensity of its expression was inversely related to tumor grade, possibly suggesting that alterations in L3 expression promote tumor progression. These findings support a role of L3 in tumorigenesis as well as in tumor progression of colon cancer. Previous studies reported that cell survival/death gene expression of Bcl-2 and Bax proteins, respectively, may be helpful in predicting clinical outcome [30, 31], patient's survival [32]. Moreover, Bcl-2/Bax ratio is implicated in the response to chemotherapeutic agents in colorectal carcinoma [33, 34]. Recently, we have demonstrated that L3 associates with CBS to shuttle it into the mitochondria for degradation. The consequent decrease in H_2_S levels correlated with cytochrome c release from mitochondria, decrease of Bcl-2/Bax ratio and caspase activation [16]. In order to clarify the biological significance of L3 elevated levels in colon tumor tissues, we evaluated the expression levels of L3 target gene Bcl-2 and Bax. In our study, a correlation between L3 and Bcl-2/Bax ratio in patients has been provided. In fact, tumor proliferation, expressed as the ratio of Bcl-2 mRNA copy number to that of Bax, was found to be inversely proportional to the decrease of L3 gene expression and increased with tumor grade, as previously reported [31] (Figure 1C). These clinical data represent the rational basis for the therapeutic targeting of L3. In fact, in the ligth of these findings we became interested to understand whether the restoration of L3 could enhance 5-FU induced colon cancer cell cytoxicity. Since most cancers lack functional p53 [19], we evaluated the cytotoxicity and apoptotic induction potential of L3 in human p53 null colon cancer cells. Previously, we demonstrated an outstanding apoptosis-inducing or -sensitizing ability of L3 in cancer cells when used at 2 μg [19]. Thus, we used this amount of L3 and a sub-toxic dose (10 μM) of 5-FU for the combined treatments. Results from MTT assay demonstrated that after treatment with L3 or 5-FU alone the proliferation rate of cells declined. Of note, the combination of L3 plus 5-FU resulted in the potentiation of the inhibitory effect of each agent on cell proliferation. Moreover, depletion of L3 made 5-FU ineffective confirming our previuos reports [15] whereas the restoration of L3 can effectively sensitize cells to 5-FU activity (Figure 2A, 2B). The colony forming ability of cells treated with 5-FU alone was partially inhibited. At day 7 of culture, about 70% of 5-FU treated cells had formed colonies, suggesting that the cells were in a quiescent state, probably dependent on 5-FU mediated cell cycle arrest. The combination of L3 plus 5-FU resulted in a more significant inhibition of the colony-forming ability, which was not restored after 7 days in culture. These data indicate that overexpression of L3 associated to the loss of clonogenic potential of the cells (Figure 2C). According to these findings, 5-FU treatment of cells overexpressing L3 significantly impaired cellular migration (Figure 2D, 2E). Since we previously demonstrated that L3 upregulates p21 expression [19], it was plausible that L3 contributed to the potentiation of inhibitory effect of 5-FU by promoting cell cycle arrest at G1 phase induced by p21. In addition, our results demonstrated that the cytotoxicity of L3 acts mainly by apoptotic cell death. In fact, the percentage of apoptotic cells detected by fluorescent microscopy after annexin V-FITC/PI staining were increased significantly following treatment with L3, suggesting that apoptosis plays an important role in the cytotoxic effects of L3 on cells. When we distinguished between early (Annexin V+ PI−) and late (Annexin V+ PI+) apoptotic cells, we found that L3 was specifically able to induce late apoptosis. Of note, the combination treatment resulted in a strong and significant increase of Annexin V positive cells (Figure 4). These results together with the notion that 5-FU sensitivity of colon cancer cells lacking p53 is dependent by L3 status in the cells [15], the concept to stimulate L3 production for a novel cancer therapy is very attractive.

Over seveal years, nanotechnology is considered crucial to promote advances in personalized medicine [35] and very promising to combine different drugs with complementary mechanisms in one delivery system allowing their localization at the pharmacological target [36]. Poor cellular uptake and rapid degradation of DNA-based therapeutics necessitate the use of delivery systems to facilitate DNA cellular internalization. Adapting nanotechnologies to the delivery of DNA has the potential to overcome extracellular barriers that limit gene therapy. Nanoparticle-based DNA delivery systema offer several potential advantages for gene delivery to cancer cells including the enhancement of gene delivery by protecting DNA from degradation and maintaining the carrier at effective concentrations, extending the duration of transgene expression [17]. To date, some findings have shown cytotoxic activity of the wild type p53-loaded nanoparticles in a breast cancer cell lines [37].

Given their ubiquity, targeted therapy using r-proteins without adversely affecting normal cells is likely to prove challenging. The selective overexpression of certain membrane receptors on cancer cells represents the opportunity for targeted delivery of cytotoxic agents that should cause strong cytotoxicity against cancer cells, with minimal effect on normal cells. HA is reported to possess strong affinity toward the CD44 overexpressing cancer cells [38]. HA is nonantigenic and biocompatible and shows high affinity toward the receptors [39]. In addition, CD44 receptors are overexpressed in different human cancer cells and their density increases with the stages of cancers [38]. Therefore, HA-based targeting delivery systems are expected to show great potential in clinical applications. In a previous study, we have developed novel polymeric nanoparticles based on a core of PLGA and a polymer shell of HA and PEI that represent a very promising system for the targeted delivery of drug combinations taking advantage of the shell and core properties [17]. In the present study, we prepared 5-FU-loaded HA-conjugated nanoparticles to target to colon cancer cells.

An important aspect to be considered while developing a therapeutic agent is its effect on normal cells. Our results show that combined NPs were not cytotoxic to normal cells and only very marginally inhibited normal cell growth as they induce cell cycle arrest (Supplementary Figure S5) suggesting the combined NPs as a possible candidate to treat human cancers. Cells treated with combined NPs exhibited efficient cellular uptake, expressed high and prolonged levels of L3 protein (Figure 6), which was functional as it induced the levels of its target gene p21 (Figure 8). Results from cytotoxicity assay show that L3 overexpression by combined NPs increase the chemosensitivity of cancer cells and are more efficient than free 5-FU in sensitizing p53 null cancer cells to 5-FU (Figure 7). We also exploited the potential of apoptosis upon treatment with combined NPs. Results from fluorescent microscopy after annexin V-FITC/PI staining documented that treatment with combined NPs was accompanied by a significant stimulation of apoptosis confirming the nature of L3-mediated pathway leading to cell death.

Increases in drug efflux are often responsible for enhanced drug resistance and are frequently due to enhanced expression of ABC (ATP-binding cassette) transporter proteins, such as P-gp [40]. It has been reported that the expression of P-gp is up-regulated in 5-FU chemoresistant colon cancer cells [22]. The failure of 5-FU treatment of HCT 116^p53−/−^ cells in condition of pL3 silencig (MTT, TMRE and clonogenic assay and [15]) prompted us to investigate whether the molecular mechanisms underlying L3-mediated enhancement of 5-FU activity involved alteration in P-gp production. Analysis of P-gp expression profile indicated that the time-dependent increased intracellular level of L3 in cells exposed to combined NPs was associated to the time-dependent decrease of P-gp intracellular amount (Figure 8). MDR1 mRNA degradation is an important cellular mechanism controlling P-gp expression that ultimately affects P-gp activity [41]. Our results show that the cancer-specific chemosensitizer effect of combined NPs may be partly dependent on L3 ability to negatively regulate MDR1 mRNA stability and consequently P-gp protein expression. Thus, the control of drug efflux through the regulation of P-gp pump expression by L3 represents a new mechanism by which this protein is albe to potentiate 5-FU activity.

Collectively, this study suggest that combined therapy of L3 plus 5-FU may help in developing therapeutic approaches for the treatment of human colon cancer lacking p53 and L3.

Finally, a proof of principle on molecular-based individualized target therapy for human colon cancer is provided.

## MATERIALS AND METHODS

### Tissues

All 30 patients of the National Cancer Institute ‘Giovanni Pascale’ of Naples gave their written informed consens according to the institutional regulations. This study was approved by the ethics committee of the National Cancer Institute ‘G. Pascale’.

### Cell cultures, DNA plasmid, transfections and treatments

The HCT 116^p53−/−^, rpL3ΔHCT 116^p53−/−^ and NCM356 cells (CVCL_D875) were cultured as previously reported [42]. The cDNA of L3 was obtained by RT–PCR from HCT 116 cells using the primers Forward: 5′-ATGTCTCACAGAAAGTTC-3′ and Reverse: 5′-TTAAGCTCCTTCTTCCTT-3′ and subsequently inserted downstream from the reporter gene GFP in the pEGFP-C1 expression vector (Clontech, Palo Alto, CA, USA) using the *EcoR*I and *Xba*I cloning sites [3]. The plasmid pEGFP-C1 (empty vector) and the fusion plasmid pGFP-L3 (pL3) were purified using QIAGEN Plasmid Mega Kit (Qiagen GmbH, Hilden, Germany). The pL3 plasmid was sequenced to verify the accuracy of the construct. Plasmid transfections were performed in cells as described [43]. Treatments of cells were performed replacing the culture medium with that containing free 5-FU or different formulations of NPs.

### RT-qPCR

Total RNA from samples was extracted from cells as previously described [44]. cDNA was synthesized from RNA as previously described [45]. The primer pairs used were: L3 (Forward: 5′-CAAAGGCTACAAAGGGGT-3′, Reverse 5′- CTCAG TGCGGTGATGGTAG-3′), p21 (Forward: 5′-TGGAGA CTCTCAGGGTCGAAA-3′, Reverse: 5′GGCGTTTGGA GTGGTAGATGGTAGAAATC-3′, Bax (Forward: 5′-CCT GTGCACCAAGGTGCCGGAACT-3′, Reverse: 5′-CCA CCCTGGTCTTGGATCCAGCCC-3′), Bcl-2 (Forward: 5′-TTGTGGCCTTCTTTGAGTTCG GTG-3′, Reverse: 5′-GGTGCCGGTTCAGGTACTCAGTCA-3′), MDR1 (Forward: 5′-ATGCCTTCATCGAGTCACTG-3′, Reverse: 5′- TAACAAGGGCACGAGCTATG-3′), β-actin (Forward: 5′ GGCGGCACCACCATGTACCCT-3′ Reverse: 5′-AGG GGCCGGACTCGTCATACT-3′). Samples were assayed in triplicate. β-actin was used as internal control for normalization.

### Mitochondrial membrane potential measurement

To quantify changes in mitochondrial membrane potential, cells were labeled with 50 nM of the mitochondrial membrane potential-sensitive fluorescent dye TMRE (Invitrogene) for 30 min a 37°C, analyzed by a Cyan-ADP Flow Cytometer (DAKOCytomation) and quantified using Summit software.

### Boyden chamber migration

Starved cells (1 × 10^5^ cells) were plated into transwell chamber in serum free D-MEM medium. The lower chambers of the plate were supplied with D-MEM medium supplemented with 10% FBS. Then, migration of cells was examined using Boyden chamber. 48 h after indicated treatments, cells were fixed and stained with crystal violet and counted under microscope.

### Clonogenic assay

Clonogenic assay was performed as previously reported [46].

### Preparation and characterization of nanoparticles

NPs loaded with 5-FU, pL3 or 5-FU/pL3 were prepared by a layer-by-layer deposition method as previously shown [17, 18]. Details of NP preparation and characterization are reported in Supplementary material S1. Briefly, a PLGA nanocore (Resomer RG502H, Evonik) was sequentially coated with PEI (25 kDa branched, Sigma), and HA (< 10 kDa, Phylcare mini HA, Bloomage Freda). 5-FU was loaded in the PLGA core while pL3 was embedded in the PEI-coated PLGA nanocore. Combined NPs were finally freeze-dried with trehalose as cryoprotectant. Fluorescent NPs (RhoNPs) were prepared analogously using a rhodamine-labelled PLGA [47] at 10 % w/w with respect to the total PLGA amount.

Hydrodynamic diameter, polydispersity index (PI) and zeta potential of NPs after each preparation step were determined on a ZetasizerNano Z (Malvern Instruments Ltd., UK).

For gel retardation assay, pL3-loaded PLGA@PEI or final NPs (10 μL, 0.5 mg/mL) were mixed with TE buffer (Life technologies) and then loaded on a 0.8% agarose gel with and without the incubation of 16 μL of DNasi (2 mg/mL). The gel electrophoresis was run at 100 V for 30 min, stained with ethidium bromide in TBE buffer (Life technologies) and observed under UV light.

5-FU and pL3 loading was assessed on freeze-dried NPs (without cryoprotectant) treated with methylene chloride and then water. The amount of 5-FU in the water phase was evaluated by HPLC while pL3 quantification was carried out by picogreen DNA kit assay (Life Technologies) after heparin addition in order to separate plasmid from complex with PEI.

Release of 5-FU and pL3 from combined NPs was assessed in DMEM FBS+ at 37°C. (See Supplementary material).

### MTT assay

MTT assay was performed as previously reported [17].

### Fluorescence microscopy

HCT 116^p53−/−^ cells were plated on coverslips at a density of 2 × 10^4^ cells per well in 6-well plates and pretreated with free HA (10 mg/ml) for 1 h before the addition of NPs. 24 h, 48 h and 72 h later, cells were fixed with 4% paraformaldehyde for 5 min. After washing, coverslips were mounted with PBS 1X-Glicerol (1:1) and stained with 4,6-diamidino-2-phenylindole (DAPI) (Vector Laboratories, CA, USA) to visualize the nuclei. Images have been acquired by using the Zeiss Cell Observer system composed by a motorized inverted microscope (Axiovert 200M) and a digital camera (Axiocam H/R). The fluorescence of was measured with Zeiss acquisition software (Axiovision 4.8.1).

### Western blotting

Western blotting analysis was performed as previously reported [48]. The membranes were challenged with anti-L3, (Primm, Milan, Italy), anti-p21, anti-Bax, anti-Bcl-2, anti-GFP, anti-Pgp, anti-β-actin (Santa Cruz Biotechnology). Proteins were visualized with enhanced chemiluminescence detection reagent according to the manufacturer's instructions (Pierce, Rockford, Illinois).

### Cell death assay

Cells were stained with Annexin V-FITC in the dark for 15 min at 4°C and then stained with propidium iodide (PI) for 5 min (BD Biosciences, USA). Cell apoptosis was quantified by flow cytometry on a BD FACSCalibur (Becton Dickinson, San Jose, CA, USA). The data were analyzed using the CellQuest Pro software package (Becton Dickinson, San Jose, CA, USA).

### Statistical analysis

Error bars represent mean ± SEM from *n* = 3 biological replicates. Statistical analysis was performed as previously reported [49].

## SUPPLEMENTARY MATERIALS


