# Supplementary material for: Enhancement of 5-FU sensitivity by the proapoptotic rpL3 gene in p53 null colon cancer cells through combined polymer nanoparticles
Source: Oncotarget. 2016 Nov 8;7(48):79670–87. doi: 10.18632/oncotarget.13216 (PMC5346744; doi:10.18632/oncotarget.13216)
Supplement: Supplementary file 1 [file oncotarget-07-79670-s001.pdf]

Enhancement of 5-FU sensitivity by the proapoptotic rpL3 gene in p53 null colon cancer cells through combined polymer nanoparticles

Supplementary Materials

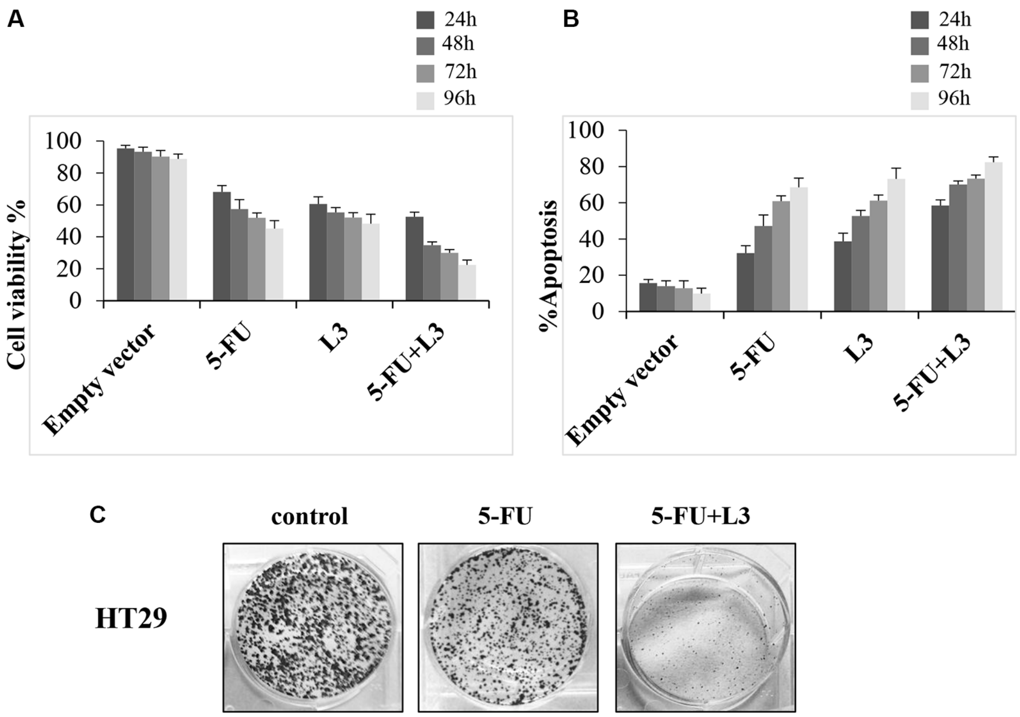

Supplementary Figure S1: Role of L3 on cell viability, apoptosis and cell proliferation in HT29 cells. (**A**) HT29 cells were transiently transfected with pL3 and treated with 10 µM 5-FU for 24 h, 48 h, 72 h and 96 h or untreated. Then, cell viability was evaluated using MTT assay. (**B**) HT 29 cells were transiently transfected with pL3 and treated with 10 µM 5-FU for 24 h, 48 h, 72 h and 96 or untreated. Then cells were analyzed for mitochondrial membrane potential by TMRE staining. Fluorescence was measured by flow cytometry. Results shown in (A) and (B) are presented as percentage (mean±SEM) (*n*=3) of the control cells set to 100% (**C**) Representative image of clonogenic analysis for cell proliferation in HT29 cells upon L3 overexpression and 5-FU treatment for 48 h. After 7 days, colonies were stained with methylene blue, photographed and counted. (**D**) Representative image of migration assay upon L3 overexpression and 5-FU treatment for 48 h. Cell migration of untreated cells was set to 100%. Results are presented as percentage (mean±SEM) (*n*=3) of the control cells set to 100%.

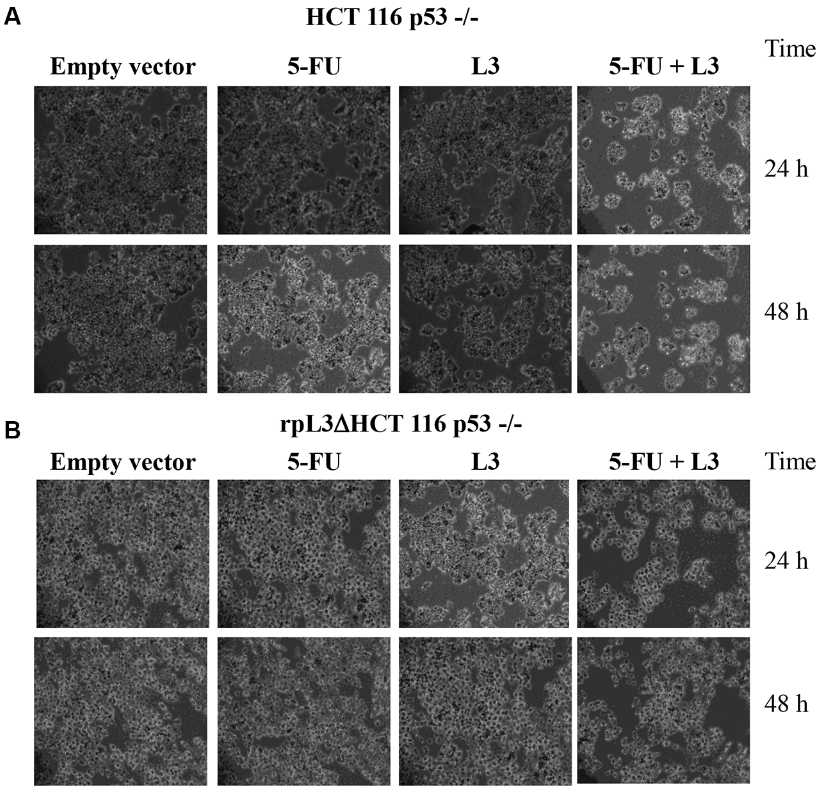

Supplementary Figure S2: Representative images of cell migration assay of (A) HCT $116^{p53-/-}$ and (B) rpL3ΔHCT $116^{p53-/-}$ cells transiently transfected with pL3, and treated with 10 µM 5-FU for 24 h and 48 h or untreated.

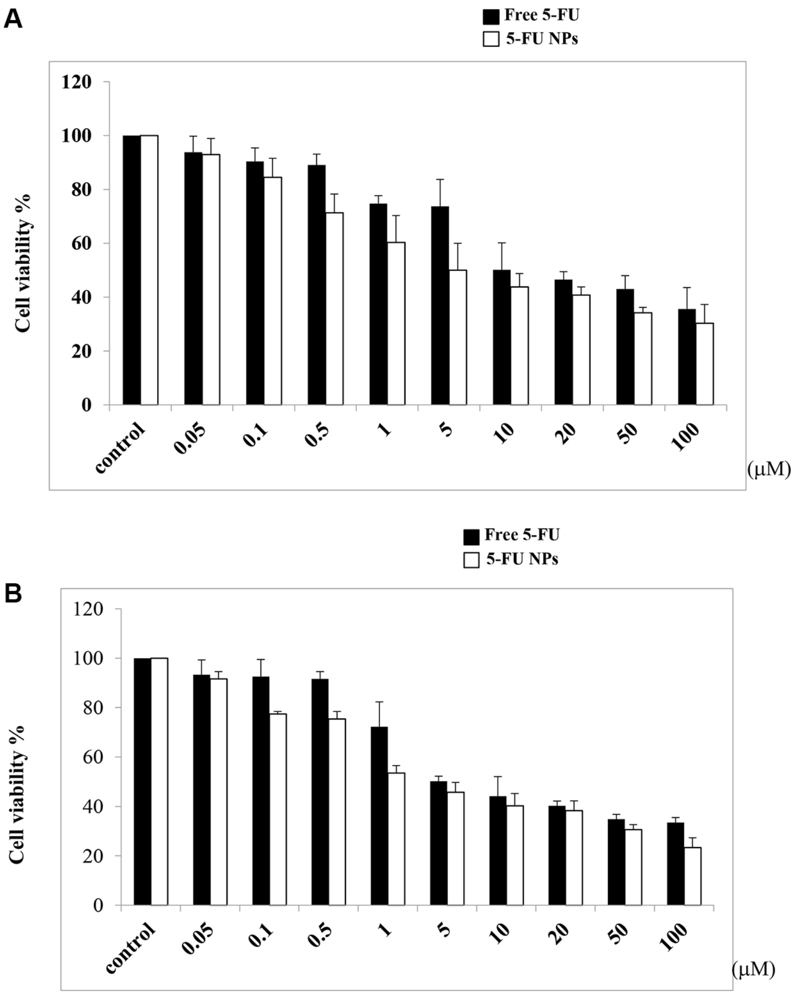

Supplementary Figure S3: *In vitro* cytotoxicity of 5-FU NPs. HCT 116p53$^{-/-}$ cells were exposed to with increasing concentrations (0.05–100 μM) of free 5-FU or 5-FU NPs for 72 h (**A**) and 96 h (**B**). After incubation, cell viability was evaluated using MTT assay. The cell viability from untreated cells was set to 100% (control). Results are presented as percentage (mean ± SEM) ($n = 3$) of the control cells.

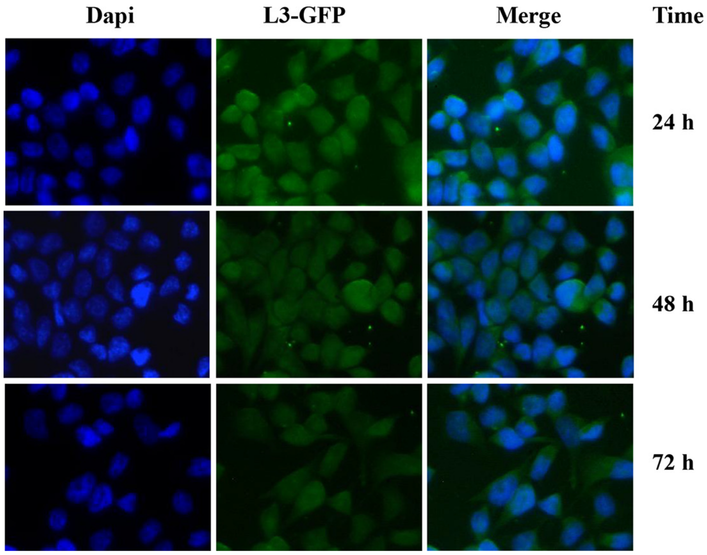

Supplementary Figure S4: Transfection efficiency of pL3H by using Lipofectamine 3000. Representative fluorescent miscroscopy images of HCT 116$^{p53-/-}$ cells transiently transfected with pL3$_H$ and Lipofectamine 3000 for 24 h, 48 h and 72 h. DAPI is used as a nuclear stain and shown in blue; L3-GFP dependent fluorescence is shown in green.

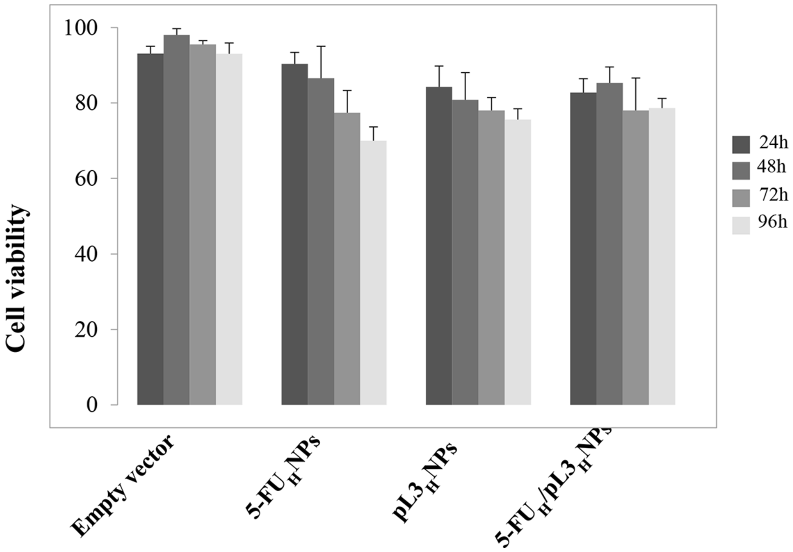

Supplementary Figure S5: *In vitro* cytotoxicity of 5-FU$_H$pL3$_H$NPs in normal colon cells. Cells were exposed to 5-FU$_H$/pL3$_H$NPs for 24 h, 48 h, 72 h and 96 h. After incubation, cell viability was evaluated using MTT assay. The cell viability from untreated cells was set to 100% (control). Results are presented as percentage (mean ± SEM) (n = 3) of the control cells.

NP preparation and characterization

NP preparation

Briefly 5-FU loaded PLGA NPs were prepared by solvent diffusion of an organic phase (2 mL) in an aqueous phase (4 mL of water with Pluronic F68 0.1%). Organic phase was prepared by dissolving 10 mg of PLGA in acetone (1.6 mL) and adding 400 µL of different stock of 5-FU in ethanol (2.5, 6, 12 and 18 mg/5 mL)in a acetone/ethanol ratio 5:1 v/v. After solvent removal under reduced pressure and room temperature, PLGA nanocore dispersion was splitted in 4 eppendorf tubes, centrifuged (5.000 g) and redispersed in water (1 mL). Thereafter, 125 µL of a PEI solution (2.5 mg/mL) was added and the PLGA@PEI nanocore centrifuged again (2.817 g) for washing and redispersed in 1 mL of water. pL3 solution in water (25 µL) was added to PLGA@PEI nanocore dispersion (0.5 mg /mL, as calculated in a separate experiment) at theoretical concentration of 10.4, 20.8 and 43.2 µg/100 µL.

Final NPs were obtained by adding 100 µL of HA in water (1 mg/mL). The interval between each addition was kept constant at 15 min. NPs were freeze-dried for 24 h with trehalose (60 mg) as cryoprotectant and kept at 4°C until use.

5-FU and pL3 actual loading

5-FU loading inside NPs was assessed by placing 0.5 mg of freeze-dried NPs (without cryoprotectant) in 500 µL of DCM and 500 µL of water. Thereafter, sample was mixed vigorously and centrifuged at 1.956 g for 5 min. The amount of 5-FU in the water phase was analyzed by HPLC on a Shimadzu apparatus equipped with a LC-10ADvp pump, a SIL-10ADvp autoinjector, a SPD-10Avp UV–Vis detector and a C-R6 integrator. The analysis was performed on a Synergy Hydro, C18 column (25 × mm). The mobile phase was a 100% (v/v) mixture of water with formic acid (99:1) pumped at a flow rate of 1 mL/min. The UV detector was set at 285 nm. A calibration curve of 5-FU in water was constructed in the concentration range 1–100 µg/mL.

pL3 loading inside NPs was evaluated by a DNA KIT ASSAY PICOGREEN (life technologies) at ex-em 480-520 nm. After NP centrifugation at 13.225 g for 5 min the pellet was treaty with DCM and water (0.5 mL) then water phase was freeze dried and redisperded in 100 µL of TE buffer 1× (10 mM Tris-HCl,1 mM EDTA pH 7.5 from life technologies) with heparin (5 mg/mL), in order to separate plasmid from complex with PEI. A calibration curve was constructed in TE buffer 1× with heparin at concentration range 0.026–1.3 µg/ mL.

5-FU and pL3 release

In vitro release of 5-FU and pL3 from 5-FU/pL3 NPs was performed on 30,05 mg of freeze-dried powder (corresponding to 0,5 mg of NPs) dispersed in 1 mL DMEM FBS+ 10% at 37°C. At predetermined time interval, the sample was centrifuged at 13.225 g for 5 min. 5-FU release was assessed in the supernatant after treatment with acetonitrile/water in ratio 1:1 and the water phase, centrifugation at 13.225 g for 5 min in order to precipitated the protein and quantify by HPLC as reported above. A 5-FU calibration curve in the range 500–5 µg/mL was constructed.pL3 release was assessed indirectly in the pellet as reported above. A pL3 calibration curve in the range 0.02–1 µg/mL was constructed.

Supplementary Table S1: Properties of PLGA core loaded with 5-FU

Formulation code[a]	5-FU theoretical loading[a] (% w/w)	Hydrodynamic diameter (nm ± SD)	P.I.	Zeta potential (mV)	Actual loading (mg/100 mg)[b]
**5-FU$_L$ PLGA**	2	124 ± 5	0.10	−31	0.15 ± 0.1
**5-FU$_M$ PLGA**	5	146 ± 5	0.12	−29	0.63 ± 0.8
**5-FU$_H$ PLGA**	10	154 ± 6	0.15	−26	0.88 ± 2.4
**5-FU$_{UH}$ PLGA**	15	170	0.11	−20	0.67 ± 0.2

[a]NP acronyms used are composed by character strings indicating: bioactive cargo (5-FU) and its theoretical loading which is 2, 5 and 10 mg per 100 mg of polymer for L, M, H and UH, respectively.
[b]Theoretical loading is expressed as initial amount of 5-FU/initial amount of 5-FU+ PLGA × 100.
SD were calculated on five different batches.

Supplementary Table S2: Properties of PEI-coated PLGA nanocore embedding pL3

Formulation code[a]	pL3 (μg/mL NPs) [b]	Hydrodynamic diameter (nm ± SD)	P.I.	Zeta potential (mV)	Actual loading (mg/100 mg)[a]
**$pL3_L$ PLGA@PEI**	2.6	166 ± 6	0.14	+35	0.15 ± 0.1
**$pL3_H$ PLGA@PEI**	5.2	158 ± 10	0.15	+33	0.36 ± 0.1
**$pL3_{UH}$ PLGA@PEI**	10.4	260 ± 2	0.30	+25	1.20 ± 0.7

[a]NP acronyms used are composed by character strings indicating: bioactive cargo (pL3) and its theoretical loading which is 2.6, 5.2 and 10.4 μg per 0.5 mg of PEI-coated PLGA NPs for L, H and UH, respectively.
[b]NP concentration is 0.5 mg/mL.
SD were calculated on five different batches.

Supplementary Table S3: Properties of final NPs containing single drugs tested in the study

Formulation codea	5-FU Act. Load. (mg/100 mg)	pL3 Act. Load. (mg/100 mg)	Hydrodynamic diameter (nm ± SD)	P.I.	Zeta potential (mV)
**$pL3_H$ NPs**	-	0.36 ± 0.1	169 ± 6	0.15	−22
**$5\text{-}FU_H$ NPs**	0.88 ± 0.1	-	178 ± 7	0.18	−23

[a]NP acronyms used are composed by character strings indicating: bioactive cargo (5-FU) and its theoretical loading which is 2, 5 and 10 μg per 100 mg of polymer for L, M, H and E, respectively.
[b]Theoretical loading is expressed as initial amount of 5-FU/initial amount of 5-FU+ PLGA × 100.
SD were calculated on five different batches.
